# Male students’ perceptions about gender imbalances in a speech-language pathology and audiology training programme of a South African institution of higher education

**DOI:** 10.4102/sajcd.v65i1.570

**Published:** 2018-05-16

**Authors:** Sandra du Plessis

**Affiliations:** 1 Department of Speech-Language Pathology and Audiology, Sefako Makgatho Health Sciences University, South Africa

## Abstract

**Background:**

The professions of speech-language pathology and audiology (SLPA) are characterised by occupational gender segregation. Reasons given by men are a lack of awareness of SLPA; a perception of poor salaries; a perception of poor working conditions; a stereotype that the profession is a female occupation; and a perception that working with children is feminine.

**Objectives:**

The aim of this study was to describe the perceptions of male students in a specific SLPA training programme regarding gender imbalances. The objectives were to describe the influences of gender on the career orientation of students, the factors shaping students’ career choices and the experiences of students during their undergraduate training.

**Method:**

This study followed a quantitative descriptive survey design. Thirty-one conveniently sampled male SLPA students, across different years of study, completed a self-administered questionnaire.

**Results:**

Participants indicated that they did not believe in the gendered nature of occupations and that SLPA are not ‘female only’ professions. They (65%) recorded that they considered salary and career prospects for choosing a career, and 74% specified that they chose SLPA because of a desire to help people. The perception of male students regarding their training appears to be positive as participants are comfortable with the clinical (71%) and learning (77%) activities. However, barriers were reported and some lecture information may be more obvious or applicable to female students. A need was identified to address the issue of working alone with paediatric clients and pertains to the societal awareness regarding sexual harassment of children, which may impact spontaneity in engagements.

**Conclusion:**

The recruitment of male students should be prioritised to better reflect the client population served by the professions. Recommendations include career counselling in rural schools, recruitment campaigns to identify interested students, public awareness regarding the professions and guidance regarding working with paediatric clients.

## Introduction

The speech-language pathology and audiology (SLPA) professions are still characterised by extreme occupational gender segregation. Globally, it is rare to come across a male SLPA in practice (Litosseliti & Leadbeater, 2013). In South Africa, no data are available on the number of registered male SLPAs and there is generally a paucity of gender studies in SLPA.

According to Litosseliti and Leadbeater (2013), there is limited research into the reasons of occupational gender imbalance as a potential key factor in career choice, despite calls to increase gender diversity in the health care professions. While young women currently may develop their potential and careers beyond the boundaries of the traditional femininity of the past, men appear to find it more difficult and are often still judged under the gender system and are expected to fit such an approach (Deutsch, 2007; Risman, 2009). Deutsch (2007) further argues for a strong focus on the elimination of the gender system to create true equality between men and women.

The development of the intersectionality theory, greatly advanced research into multiple forms of oppression in different social settings and became indispensable in the analysis of global gender inequalities (Alinia, 2015). Intersectionality highlights the fact that discrimination may overlap; for example, racial and gender discrimination may occur simultaneously in the workplace or in other arenas of life. Although the intersectional theory advanced research on African women globally, Peretz (2017) in his intersectional analysis of men’s pathways found that not only will our understanding of men improve by attending to men’s intersecting identities, but also our understanding of men as allies to women’s rights movements.

In SLPA, there appears to be no prospect of redressing the gender imbalance in the near future (Boyd & Hewlett, 2001). Male speech-language therapists (SLT) in the Royal College of Speech and Language Therapy’s continuing professional development network indicated why men do not choose SLT as a profession. McKinson (2007) lists five reasons why men eschew a career in SLPA, including a general lack of awareness of the profession, perceptions of costly training, poor salaries and poor working conditions, and the stereotype that this is a female occupation.

Client caseloads suggest that more male SLPAs would enable these professions to better reflect the population they serve as the majority of paediatric and adult clients are males. Many different theories have been formulated to support the matching of therapists and clients on the basis of gender with the premise that people identify and understand people similar to themselves better. Such shared understandings are thought to build stronger therapeutic relationships with more successful therapeutic outcomes (Bhati, 2014).

Recruitment of more men into the profession requires addressing gender stereotypes. Gender inequality in SLPA appears entrenched and it is difficult to envisage how gender balance could be achieved. While a substantial body of research on women in male-dominated professions is available, fewer studies examine the experiences of men in female-dominated professions (Budig, 2002). This article explores male SLPA students’ perceptions, regarding the influences of gender imbalances in career choices, career opportunities, learning experiences and related challenges.

## Literature review

### Career orientation

The industrial revolution of the 1750s resulted in the establishment of large-scale factories in which machines replaced handcraft in the manufacturing process, thereby leading to the division of tasks along male- and female-dominated fields. Jobs were subdivided by allocating tasks that required more technical skills to use the new technologies to men and more routine and repetitive tasks to women (Hall, 2005; Litosseliti & Leadbeater, 2013).

While health care was mostly provided by women to family and friends in the pre-industrial times, the medical profession established itself as male-delivered for financial reward during the industrial revolution. The cures of the midwives and the untrained healers were discredited. During this time only middle and upper class men accessed male-dominated universities, while women, although not excluded from writing the examinations, were not allowed to attend the university classes. A small number of wealthy women passed the examinations after they found alternative approaches to studying (Witz, 1992). In this patriarchal society, men therefore enjoyed gender privilege and dominated the medical profession.

While society recognised the role of women in the private sphere of the home during the 19th century, the last few decades of that century showed change as women started to enter the workforce. Professions such as nursing were highly recommended for women because these female-dominated jobs were perceived to demand more ‘emotional labour’ – the effort employees put into managing others’ and their own emotions (Hall, 2005, p. 189).

In the English-speaking sub-Saharan African countries, the informal sector became an important socio-economic and political phenomenon during the past three decades. While some black African women improved their economic status after entering this sector, it caused greater burdens for others. Some women experienced discrimination in terms of lack of access to credit, lack of access to property, insufficient capital, as well as police harassment. In addition, women encountered domestic violence as men felt threatened by women’s financial independence and their empowered roles in decision-making (Ampofo, Beoku-Betts, Njambi, & Osirim, 2004).

Over the past few decades, the number of students in higher education in industrialised countries has risen partly because of the enrolment of more female students. However, as professions evolve, conflict is still evident in terms of gender issues, and student recruitment in certain professions is still presenting a gendered imbalance. It appears that students who enrol in gender-traditional professions often had an early preference for these programmes. Notwithstanding this, the choosing of gender non-traditional programmes by students is increasing among both female and male students (Mastekaase & Smeby, 2008).

Mastekaase and Smeby (2008) provided three explanations for gender segregation in career choice: firstly, the nurturing role of women may encourage them to choose caring occupations; secondly, women may choose professions in which it is easier to combine employment and family life; and, thirdly, men and women may be exposed to gender discrimination (usually from outside the professions) and therefore decide against certain professions. In addition, Dick and Rallis (1991) reported that remuneration and career advancement were usually more important factors in career choice for men. Bai (1998) found that the market economy (the basis of the capitalist system) changed the values of university students in that they put self-interest before societal interests and rate money and power as the primary motivators for finding a job. Furthermore, a profession’s perceived value is also determined by intrinsic factors such as intellectual interest and the desire to help people, as well as extrinsic factors such as salary expectation and the cost and length of future training (Bai, 1998).

While excellent progress has been made by women in previously male-dominated professions, such as business and medicine (Ross & Coleman, 2008), men in female-dominated fields do not necessarily have it easy. They report hostile behaviour, not from colleagues but from friends. They also report anxiety caused by a mismatch between stereotypical masculinity and working in a female-dominated field. Men in these fields downplay the feminine aspects of their jobs and accentuate the masculine aspects (Ross & Coleman, 2008). In addition, men who choose female-dominated professions are often perceived as having degraded themselves in status (Lassen, 2012). It appears that this perception, which is fortified by cultural and political understandings of acceptable gender roles, has dominated for men (Ross & Coleman, 2008). Therefore, the following question arises: why men do pursue careers in female-dominated professions and specifically in SLPA?

### Factors that affect career choices

The training of SLT and audiologists is closely related, as both professions engage with individuals’ communication difficulties. Historically students were trained in combined programmes as both SLT and audiologists, but currently only single combined programmes are still offered in South Africa, while the majority of the institutions of higher education offer separated programmes.

Speech-language pathology and audiology are regarded as small professions in countries such as South Africa, New Zealand and Australia (Byrne, 2010). Although the statistics for male SLPAs in South Africa are not available, Litosseliti and Leadbeater (2013) reported that male SLTs are found in the following percentages in the selected countries: 2.5% in the UK, 3% in Australia and 4.1% in the USA.

In 2015 and 2017, respectively, 106 and 130 male students were registered at the various institutions of higher education in South Africa for SLPA (Table 1).

**TABLE 1:** Demographic information of all male undergraduate students of all year levels registered at various institutions of higher education in South Africa for speech-language pathology and audiology in 2015 and 2017.

Institution of higher education	Programme offered	Number of male students per university in 2015	Male students per university in 2015 (%)	Number of male students per university in 2017	Male students per university in 2017 (%)
Sefako Makgatho Health Sciences University	Speech-language pathology and audiology	37	37	50	40
University of Cape Town	Speech-language pathology or audiology	36	11	31	9
University of KwaZulu-Natal	Speech-language pathology or audiology	19	9	35	14
University of Pretoria	Speech-language pathology or audiology	8	3	6	2
University of the Witwatersrand	Speech-language pathology and audiology	3	2	3	2
University of Stellenbosch	Speech-language pathology	3	2	5	4

Note: The information was gathered at the Interuniversity Head of Department meeting on 27–28 August 2015 at Cape Town and 21–22 August 2017 at Johannesburg.

Comparatively, the smaller percentage of male students (Table 1) could be because of factors consistent with those reported in the literature. Farmer (1985) explains that career choices can be linked to three interactive influences: background (e.g. gender and ability), psychological or personal factors (such as attitudes, beliefs and earlier experiences) and environmental or cultural factors (e.g. norms, peer pressure and media influences). A study by Agarwala (2008) showed that fathers exerted the greatest influence on the career choice of students in India, for both male and female students. For female students, the second most important influence was their mothers, and for male students, the second most important influence was their friends. Research results cited by Mastekaase and Smeby (2008) indicate that women favour support by parents in the choice of careers and are more likely to be influenced by peers, while contrary to the findings in the previous study, men tend to be more resistant to parental involvement. Dick and Rallis (1991) reported that parents and teachers were perceived to be the major influences on career choice, more often for students (both men and women) choosing careers in engineering and science.

Litosseliti and Leadbeater (2013) conducted a study including face-to-face interviews with new graduates and practising qualified SLTs about factors contributing to their career choice and found four main factors. These included: (1) exposure to SLT, being it through personal experience or a family member receiving therapy or through direct contact and encouragement from a relative or career advisor; (2) the influence of parents and relatives; (3) the perceived status, prestige and potential salary; and (4) personal motivation and interest in the selection of a career in SLT.

### Opportunities and challenges male students experience during teaching and learning in the health care professions

In health care sciences training programmes, such as nursing and occupational therapy, more male students are entering these female-dominated fields and this raises the question whether students’ gender has implications for training. Male nurses have expressed the frustration that they are assumed to be gay and also merely seen as muscle power to carry out heavy work (Parish, Carr, Suwinski, & Rees, 1990). Male students in occupational therapy reported that female patients have refused therapy related to the field of dressing practice. These students also saw the craft activities as female orientated and indicated that they get ‘mothered and smothered’ by female staff members and students (Parish et al., 1990, p. 69). Smedley (1998) refers to the problems in recruiting male student teachers as a feminised curriculum, feminised assessment regimes and feminised assessment methods. In SLPA there is a challenge for male students working alone with paediatric clients (Boyd & Hewlett, 2001). This pertains to the societal awareness and caution regarding the sexual harassment of children, which may have an impact on spontaneity when engaging with young clients (Boyd & Hewlett, 2001). The results obtained by Litosseliti and Leadbeater (2013) indicated an expectation that male therapists should rather work in specialities such as adult neurology.

Although the literature revealed some challenges for male students, there is limited data available on the advantages of being male in a female-orientated programme. In the research by Litosseliti and Leadbeater (2013), male SLTs as well as lecturers indicated that participants perceived that in terms of career progression and promotion opportunities, qualified male therapists progress faster than their female counterparts. They explained the large number of men in management positions by speculating that men make better managers because they are perceived to be more rational than women who are more emotional.

The current research project wanted to describe male SLPA students’ perceptions regarding the influences of gender in career choices, the factors shaping career orientation, learning experiences and related challenges in order to serve as an introduction of the topic in South Africa.

## Methodology

The research project set out to describe the perceptions of male students in an SLPA training programme in a South African institution of higher education regarding gender imbalances in career choice in a female-dominated programme. In particular, from the key areas outlined above, the following three objectives were formulated:

- to describe the influence of gender on the career orientation of male students
- to describe the factors shaping male students’ career choices
- to describe the experiences of male students during their undergraduate training.

### Research design

A descriptive survey design implementing the quantitative perspective was selected for the purpose of the research project. In the descriptive survey, data were obtained through questionnaires and limited open-ended questions were included. The research outcomes could not be extended to other groups and generalisation beyond the research context was not possible.

### Participants

Male students were identified from class lists. Out of 43 conveniently sampled undergraduate SLPA male students of 2016 in a South African institution of higher education, 31 consented, completed and returned the questionnaires.

The demographic information of the students who gave consent to participate in the research shows a spread of young adult male students between the ages of 18 and 29 (Table 2). Some of the students being in their late 20s may have indicated that they commenced their studies in SLPA at a later age or may have completed other training programmes before entering the programmes. Another factor to consider in South Africa is that many students, because of financial constraints, may have had to work to save money before they can enrol in a university.

**TABLE 2:** Demographic information of the participants (*n* = 31).

Characteristics	Description	Number of participants	%
Age	18–20	16	52
	21–23	9	29
	24–26	2	6
	27–29	4	13
Race	African	31	100
Nationality	South African	29	94
	Other	2	6
Home area	Urban	14	45
	Rural	17	55
Year of study	First year	15	48
	Second year	6	1
	Third year	6	19
	Fourth year	4	13

It is interesting to note that all of the male students in the first-year class took part in the research. It is also positive to note that the number of participants from second to fourth year constitutes more than 50% of the responses, providing a balance in the perceptions of younger and more senior students.

### Material

The survey questionnaire was designed by the researcher as other tools were not specifically accurate for this study. The questionnaire content was developed after a literature review and was mainly based on earlier relevant research on male perceptions regarding the SLPA and nursing professions by Anderson (2014), Litosseliti and Leadbeater (2013) and Boyd and Hewlett (2001). The questionnaire was divided into four sections:

- Section A: Demographic information
- Section B: Influences on career choice
- Section C: Career orientation towards traditional and non-traditional occupations
- Section D: Opportunities and challenges that male students experience in teaching and learning.

In Section A, participants were requested to respond to a set of four personal questions. In Sections B, C and D, the participants were asked to respond according to the Likert scale. There were two sets of response options. The first set of questions included options relating to agreement, namely, *strongly disagree, disagree, agree,* and *strongly agree.* The second set of questions included options relating to frequency, namely, *never, sometimes, mostly* and *always.*

Participants were further requested to respond on the dichotomous scale to ‘yes/no’ questions and additional questions were also included to allow for clarification of responses where necessary. Some counter-test items were included in the questionnaire to increase the validity of the participants’ responses. Open-ended questions were included at the end of each section to allow participants to elaborate on the topic.

A pilot study was conducted to assess the need for adaptations in the procedures or wording of the questionnaire. Four male students (one per year level) from another institution of higher education in the speech-language pathology or audiology programmes formed the trial group of pilot study participants. During structured interviews, the pilot study participants approved the questionnaire and made no recommendations for change. This improved the face validity of the questionnaire.

### Data collection

Permission to access the students was granted from the university and the Department of Speech-Language Pathology and Audiology, and ethical clearance was obtained. The researchers met with all the participants in one setting, as a group, to explain what the questionnaire entailed and what would be expected from them. Interested participants completed the consent forms and proceeded to completing the questionnaires given to them as a hard copy. The researchers were available during the completion of the questionnaires to provide clarity where necessary.

### Data analysis

In the analysis of results, the Likert scale responses were collapsed to make it more manageable and strongly disagree and disagree constituted disagree, while agree and strongly agree constituted agree. Data were entered onto an Excel spreadsheet. Data analysis was repeated to improve reliability.

Descriptive statistics (Leedy & Ormrod, 2005) were mostly utilised to describe the data and to illustrate trends within the research context. Descriptive statistics used included frequencies and percentages to analyse quantitative data. Qualitative data were manually organised into immerging categories or subcategories based on similarities (De Vos, 2002).

## Ethical consideration

The research project was approved by the Sefako Makgatho University Research Ethics Committee (SMUREC/H/59/2016:UG). Ethical considerations of protection from harm, informed consent, right to privacy and honesty with professional colleagues were considered throughout the project.

## Results and discussion

### Influence of gender on career orientation

In the questionnaire male students were requested to indicate on a Likert scale the influence of gender on career orientation. More than half of the students (58%) indicated that they did not believe in the gendered nature of occupations and most of the students (65%) also did not perceive that gender should be considered when choosing a career. The difference of opinion, and the large number of students not affected by gender difference in their professions was unexpected. It is speculated that these students observed a gender mix in the health care professions on the campus of their place of study with which they could associate. In addition, the students may not have been exposed to the wider work environment and may not have experienced gender segregation in professions. The opposite was found by Francis (2002) in her research with high school learners, which indicated gender dichotomy with girls still opting for the caring professions and boys choosing occupations where business, scientific and technical skills are required.

The majority (68%) of participants indicated that both genders were affected by inequalities in the workplace, but only about half of the students (48%) thought that their gender would affect their future promotions. Some unfavourable conditions often referred to as the ‘glass escalator effect’ and the ‘revolting door effect’ are experienced by women in male-orientated professions and vice versa. This refers to conditions of inequality in the workplace created by occupational gender segregation that adversely affect male as well as female professionals (Litosseliti & Leadbeater, 2013).

The large majority of students (74%) indicated that the word ‘therapist’ does not remind them of a woman and they feel comfortable to be addressed as such. This contradicts Litosseliti and Leadbeater’s (2013) findings where SLT students, SLT lecturers, career counsellors and qualified SLTs indicated that the words ‘therapist’ and ‘therapy’ generally have a female connotation. In all four of these groups, participants indicated that the words ‘therapist’ and ‘therapy’ are in general female orientated.

Students were divided over the statement that women are natural communicators as 48% disagreed. It is postulated that students may know about the common perception that girls and boys differ from childhood, with girls performing well in communication skills and boys in manual skills. However, Litosseliti and Leadbeater (2013) agreed that the general perception is that women are better communicators as found in their study. It was also noticed that there may be a bias against male students in the selection of students to enrol into an SLPA programme as a result of this perception.

Interestingly, the majority of students (65%) indicated that even though they consider salary and career prospects when choosing a career, 74% indicated that they chose SLPA because of a desire to help others. This is very positive as it is generally known that there is a dire need for caring professionals to serve and care for the underserviced communities in South Africa. On the other hand, it is also the right of a qualified therapist to earn a living wage and to have a career path ahead that includes promotions for work well done.

The majority (97%) of students indicated that the careers (SLPA) are not only for women and therefore not female professions. Litosseliti and Leadbeater (2013) indicated in their research that the career counsellors were the specific group of participants that considered them female professions. This finding has implications for the marketing of the professions where a more realistic view should be presented to prospective students and learners, especially by career counsellors who may influence the career choice of a prospective candidate.

Students were requested to complete three open questions regarding gender distribution in the department. Categories that emerged as well as selected verbatim responses are presented in Table 3.

**TABLE 3:** Gender distribution in the specific department.

Areas	Categories	Examples of verbatim responses
Expectations regarding gender distribution in the programme	Expected more women than men in the programme (*n* = 7)	‘Obviously more women than men. But I did not expect so many males in class.’
	Expected more men than women in the programme (*n* = 2)	‘I thought males will be dominant.’
	Expected a balance in gender distribution (*n* = 11)	‘I was expecting a balance in gender distribution. Almost equal males and females. I was sadly mistaken.’
Feelings regarding gender imbalance in the Department of Speech-Language Pathology and Audiology	There are no concerns regarding the gender imbalance (*n* = 7)	‘Everything is going the same between the males and the female.’
	There is disappointment about the gender imbalance (*n* = 7)	‘Disappointing, since females are the ones dominating course.’
	It is expected from the department to enrol more males in the profession to balance the genders (*n* = 7)	‘It’s insufficient. We need more males in the department.’
Reconsidering career choice because of gender imbalances in the profession	Did not consider to change career (*n* = 15)	‘I believe working with patients in a health care does not have anything to do with gender.’
	Did consider career change (*n* = 3)	‘It is too female-dominated.’

The students in the research context were divided according to their expectations regarding the gender distribution in the programme before they entered into the programme (Table 3). As the global tendency is that the professions are female-dominated (Litosseliti & Leadbeater, 2013), these results are rather unexpected. It raises the question whether some of the students have been in any contact with an SLPA at all before entering the programme and may be indicative that they have not encountered an SLPA because of the limited numbers of qualified SLPAs in South Africa, or the unavailability of services in the rural areas or even the fact that the professions are unknown to a large majority of local population.

The results further indicate a general feeling of disappointment for the students regarding the gender distribution and an expectation that more male students need to be recruited (Table 3). In Litosseliti and Leadbeater’s (2013) study, the need to prioritise male SLPA students’ recruitment was reported. Boyd and Hewlett (2001) reported that the existing low number of men in the professions is probably the main factor discouraging other men to be recruited into the professions. McKinson (2007) suggests to re-brand the image of the profession and to provide positive images of male SLTs in the media.

Although many students (15) did not consider a change in career as a result of the gender representation, a small but alarming number of students (3) considered changing their career choice as a result thereof (Table 3). Parish et al. (1990) found in their research that 28% of male occupational therapists contemplated leaving their profession as they felt dissatisfied with the career potential for male therapists in their profession. This emphasises not only the need for support of the male students in the programme, but also the need for appropriate career counselling to allow students to make informed decisions regarding something as important as a future career.

### Factors shaping career choices

In the questionnaire male students were requested to indicate on a Likert scale (relating to agreement) the factors shaping their career choices. Less than half of the students (48%) indicated that they knew what the profession entailed before enrolling. It is alarming that the other students enrolled without making informed decisions regarding their future career. It is speculated that SLPA are not well-known professions among some African communities, especially in the rural areas of South Africa. This may account for this response, as 55% of the students were from rural areas (Table 2).

Eighty-four per cent of the students indicated that SLPA was not their first-choice career. These students therefore applied for other programmes as first choice and considered SLPA as an alternative. They enrolled in the programme after not being selected into their first-choice careers. These students, therefore, are not motivated and passionate about a career in SLPA and may drop out of the programme without completing it or alternatively re-applying for their first choice after a year in the programme or on completion of the programme.

Although SLPAs add significant value to the quality of life of children and adults with speech, communication, language, swallowing and hearing disorders, it is clear from the literature (Byrne, 2010) that there is a general lack of awareness among the public about these professions. This may have an impact on the choice of students to enter the profession of SLPA.

Although 87% of students did not have any kind of contact or experience with SLPAs, they selected this as their future career. Research by Litosseliti and Leadbeater (2013) found that experience with an SLT was a common factor that influenced the choice of the career for students, therapists and lecturers. Byrne (2010) found that the most influential factor that creates a desire to become an SLT was to have contact with a therapist and understand what the profession entails. However, individuals who have received intervention themselves are less likely to enter the professions because of the experience and perception created through this encounter.

Interestingly, the majority of students indicated that they were not influenced by their parents (77%), teachers (81%) or media exposure (84%) to choose a career such as SLPA. If the students were not influenced by their parents, teachers or the media, where did they learn about the professions? In an open question, the students indicated that they learned about the professions during the process of late application (25%), from the university prospectus (19%), from community members at home (19%), from the Internet (16%), from current students (16%) and from therapists at their local hospital (5%). These results provide an indication of the importance of word of mouth in the marketing of the professions of SLPA as not all prospective students may have access to Internet in South Africa.

### Experiences of male students during undergraduate training

In the questionnaire the male students were requested to respond on the dichotomous scale to ‘yes/no’ questions regarding their experiences during undergraduate training. The results are presented in Table 4.

**TABLE 4:** Perceptions regarding the experiences during undergraduate training (*n* = 31).

Statement	Disagree		Agree	
	No	%	No	%
I feel nervous about learning or practising clinically.	22	71	9	29
I feel uncomfortable with some learning activities.	24	77	7	23
As a male student, I feel excluded from groups or learning activities.	29	94	2	6
I can relate to the examples in class.	3	10	28	90
The activities are female orientated.	23	74	8	26
I am treated differently from female students.	29	94	2	6
Male students are dominant in class discussions.	25	81	6	19
I have encountered examples and discussions that seemed more applicable to women than men.	18	58	13	42
Lecturers change teaching examples to highlight information for the male students that would have been more obvious to the female students.	20	65	11	35
The lecturers are aware of barriers that may exist for male students in SLPA.	16	52	15	48
There is a need for a separate set of guidelines for male students.	24	77	7	23
I received guidance on working alone with children.	17	55	14	45
Male students are more aware than female students of issues around child abuse.	15	48	16	52
I prefer working with adults rather than children.	14	45	17	55
I experience difficulties as a man from the attitude of clients.	22	71	9	29

SLPA, speech-language pathology and audiology.

The general perception of the male students regarding their experiences during undergraduate training appears to be positive as the majority of students were comfortable with both the clinical (71%) and learning (77%) activities. They could relate to examples in class (90%) and did not experience the activities as female orientated (74%) (Table 4). The latter fact contradicts findings in other female-orientated professions. Parish et al. (1990) found that their participants experienced the occupational therapy curriculum as female orientated and in the research by Mills, Martino and Lingard (2004) male teachers reported female curricula in teacher education.

However, some students found examples (42%), information (65%) and barriers (52%) in teaching and learning more obvious or applicable to female students and indicated that lecturers were unaware of this. It is speculated that the lecturers may be incognisant of the situation or alternatively unsure of how to handle these areas of teaching and learning. A need is therefore identified to alert lecturers about these perceptions of the students. Parish et al. (1990) suggest that appropriate guidelines for both students and lecturers need to be developed as there is an indication that gender influences some of the activities undertaken.

Students were divided regarding the important issue of working with paediatric clients. The results indicated that 55% of students did not receive any guidelines on working alone with children, 52% of students responded that they were more aware about child abuse than female students, while 55% preferred working with adults rather than working with children (Table 4). Although there was no extreme positive or negative opinion from the students, there is a need identified that this matter should be addressed with the students. Literature (Boyd & Hewlett, 2001; Litosseliti & Leadbeater, 2013; Parish et al., 1990) highlights that a set of guidelines should be identified and formulated for male students and lecturers regarding working with children. Such guidelines may also help to prevent male professionals from dropping paediatric clients from their caseloads once qualified.

The students were requested to complete open questions regarding the advantages of being male in a female-orientated training programme. Four categories of advantages of being male in a female-dominated training programme were analysed (Table 5). Selected quotes from the participants are used to illustrate each subcategory.

**TABLE 5:** Advantages of being male in a female-orientated training programme (*n* = 24).

Category	Subcategory	Examples of verbatim responses
Neutral (*n* = 4)	No advantages	‘There are no advantages.’
Self-esteem (*n* = 6)	Less competition	‘Not too much competition among males and that increases my self-esteem.’
	Special treatment	‘You are treated special and trained with good supervision.’
	Increased confidence	‘Confidence and bravery in practice.’
Knowledge (*n* = 5)	Increased knowledge about women and children	‘I get to understand females and children.’
	Learn to link theory to practice	‘Learn more and associate theory with practicals.’
Benefit of being male (*n* = 9)	Execute duties that women cannot	‘I am able to do certain things that females cannot do like lifting heavy duty material and I am able to participate more physically in activities than females.’
	Perceived as more competent than women	‘You are confused for a medicine student, you seem superior to other female students during clinicals.’
	Children seem more comfortable with men	‘Children (both male and female) seem more comfortable with males 80% of the time.’
	Men work faster with clients than women	‘I can work fast with patients.’
	Eliminate stereotyping	‘To curb stereotyping and account for lack of male professionals.’
	Less attached to clients	‘You don’t emotionally attach easily to clients, so there is no deep feelings and emotional breaks.’

An interesting category that emerged is the benefits of being male. While the students in the current study perceived their stronger physical abilities as positive and were willing to carry heavy objects around, participants in the study by Parish et al. (1990) were negative about this and indicated that they perceived that they were merely seen as muscle power to carry out heavy work.

A subcategory that emerged is that some male students were of the opinion that it is an advantage to be less attached to clients. This correlates to research by Williams (1992), where male teachers indicated that they conscientiously remove themselves from the ‘mothering’ or emotional aspects when working with children and adopt a more neutral approach in interaction. It is indeed promising that the students in the current study realised that although they need to care and serve their clients, emotional involvement need not be overwhelming.

In a further open question students indicated the challenges of being male in a female-orientated training programme. The categories and subcategories that emerged from the data analyses are summarised in Table 6.

**TABLE 6:** Challenges of being male in a female-orientated training programme (*n* = 22).

Categories	Subcategory	Examples of verbatim responses
Neutral (*n* = 2)	No challenges	‘I have not yet encountered any challenges.’
Gender-based challenges (*n* = 15)	Working with children	‘Playing with children during sessions.’
	Being surrounded by women	‘Surrounded by females.’
	Being creative	‘Can’t really think of creative activities.’
	People judge you	‘Being judged based on what you do, without people considering the significance you make in people’s lives.’
	Behavioural changes	‘You get to face thing that you have never seen before in your life time and you are forced to adapt and behave like a female in certain occasions.’
Female advantages (*n* = 5)	Unequal treatment from women	‘Treated differently from females.’
	The course is female orientated	‘Most of the things are based on females.’
	Children prefer working with women	‘Many children prefer females.’

Many students (68%) who responded to this question experienced gender-based challenges in the educational setting (Table 6). The fact that working with children needs to be addressed by the professions is noted once again. Interestingly, some of the male students find it a challenge to be surrounded by women. The reason can be twofold as students may view the ‘mothering’ by some staff and fellow female students in a negative light as discussed earlier, but it may also be culturally difficult for African male students to be taught in a department with only female lecturers and supervisors. African culture is generally male-dominated, with woman acting submissively resulting in an unequal power relation between the genders (Ngubane, 2010). Therefore, it may be a challenge to find something outside the norm as seen in the SLPA programme in the research context.

Some students indicated that they find it difficult to be creative (Table 6). Creativity is inherent to SLPA, especially when working with children. This may be one of the reasons why some students prefer working with adults (Table 4) as some of the activities with children may be perceived as female orientated. These results correlate with the results of Parish et al. (1990), which indicated that the occupational therapy students experienced the craft activities as such.

It is a concern that the students perceive that they have to change their behaviour and adopt ‘female behaviour’ in certain situations during their training. This indicates that gender definitely influences some of the activities undertaken. This result clearly indicates that the teaching and learning activities need to be transformed to make it appropriate and acceptable for male students.

## Summary and conclusions

To integrate both men and women in all professions does not only require the removal of barriers for women, but also includes the dismantling of barriers that men have to overcome in female-dominated professions such as SLPA.

In general, it is clear that there is great potential for male professionals in SLPA and that men can add significant value to the intervention with both adults and paediatric clients. However, public perception, recruitment, work experience as well as career development need to be addressed in order to motivate men to join these professions.

In summary, the following recommendations are drawn from the research:

- The need for career counselling in rural schools in South Africa was identified in order to empower learners with knowledge before making important decisions regarding future careers.
- The need for recruitment campaigns was identified in order to select prospective students who really are interested in a career such as SLPA.
- Public awareness about the professions and the scope of practice need to increase, which may have benefits that are twofold: firstly, for the profession as appropriate referrals will increase access to services and, secondly, more people may act as resources and potentially recommend SLPA as a career path for interested students.
- There was a need identified that the matter regarding working with paediatric clients should be addressed with the students and that a clear set of guidelines should be compiled in order to protect students and clients from any unpleasantness.
- The students’ experiences reveal that transformation of the teaching and learning activities are needed to make it appropriate for male students.

### Limitation and strength

The current study was an exploratory study and the findings cannot be presented as indicative of all male students in all contexts in South Africa as they represent only the opinions of a specific group of students. Although structured interviews could not be carried out because of time limitations, the questionnaire produced many spontaneous comments, thus increasing the depth of the survey. The current study is seen as an introduction to the topic in the South African context and needs to be explored through further research.
